# *rac*-1-(4-*tert*-Butyl­phen­yl)-5-ethyl-4-ferrocenyl-5-hy­droxy-1*H*-pyrrol-2(5*H*)-one

**DOI:** 10.1107/S2056989023001858

**Published:** 2023-03-07

**Authors:** Tobias Biletzki, Helmar Görls, Wolfgang Imhof

**Affiliations:** a University Koblenz, Institute of Integrated Natural Sciences, Universitätsstr. 1, 56070 Koblenz, Germany; b Friedrich-Schiller-University Jena, Institute of Inorganic and Analytical Chemistry, Humboldtstr. 11, 07743 Jena, Germany; Universidad Nacional Autónoma de México, México

**Keywords:** crystal structure, pyrrole, pyrrolone, ferrocene

## Abstract

The title compound, [Fe(C_5_H_5_)(C_21_H_24_NO_2_)], which is produced by the oxidation of 1-(4-*tert*-butyl­phen­yl)-2-ethyl-3-ferrocenyl­pyrrole, crystallizes as a racemic mixture in the centrosymmetric space group *P*2_1_/*n*. The central heterocyclic pyrrole ring system subtends dihedral angles of 13.7 (2)° with respect to the attached cyclo­penta­dienyl ring and of 43.6 (7)° with the major component of the disordered phenyl group bound to the N atom. The 4-*tert*-butyl­phenyl group, as well as the non-substituted Cp ring are disordered with s.o.f. values of 0.589 (16) and 0.411 (16), respectively. In the crystal, mol­ecules with the same absolute configuration are linked into infinite chains along the *b-*axis direction by O—H⋯O hydrogen bonds between the hy­droxy substituent and the carbonyl O atom of the adjacent mol­ecule.

## Abstract 2

The title compound,which is produced by the oxidation of 1-(4-*tert*-butyl­phen­yl)-2-ethyl-3-ferrocenyl­pyrrole, crystallizes as a racemic mixture in the centrosymmetric space group *P*2_1_/*n*. In the crystal, mol­ecules with the same absolute configuration are linked into infinite chains along the *b-*axis direction by O—H⋯O hydrogen bonds between the hy­droxy substituent and the carbonyl O atom of the adjacent mol­ecule.

## 1. Chemical context

In a series of recent publications, we were able to show that the ruthenium-catalysed four-component reaction of an α, β-unsaturated aldehyde with a primary amine (producing an inter­mediate imine), carbon monoxide and ethyl­ene produces a library of chiral 1,3-di­hydro­pyrrolo­nes and pyrroles, respectively (Biletzki & Imhof, 2011 ▸). The ratio of those two products is highly dependent on the relative permittivity of the solvent used, with the yield of the pyrrole increasing with the polarity of the solvent (Gillies *et al.*, 2007 ▸). We were also able to show that the oxidation of the resulting pyrroles with oxygen leads to the formation of derivatives of the title compound (Dönnecke & Imhof, 2003 ▸). There are some similar reactions reported in the literature where a pyrrole was transformed into a hy­droxy-pyrrolone by oxidation with O_2_, but the reaction mixture had to be irradiated in the presence of a photosensitizer, or radical initiators such as AIBN had to be added in order to induce the reaction (Machida *et al.* 1982 ▸; Dannhardt & Steindl 1985 ▸, 1986 ▸; Takechi *et al.* 1988 ▸; Boger & Baldino 1991 ▸; Procopiou & Highcock 1994 ▸; Gonzalez *et al.* 1999 ▸). Therefore, a radical mechanism cannot be ruled out for the formation of the title compound, although no addition of any typical initiator is necessary. So overall, depending on the reaction conditions, either chiral 1,3-di­hydro­pyrrolo­nes, chiral 5-hy­droxy-1,5-di­hydro­pyrrolo­nes or 2,3-disubstituted pyrrole derivatives might be the main products of the catalytic synthetic methodology developed in our lab (Biletzki & Imhof, 2011 ▸; Gillies *et al.*, 2007 ▸; Dönnecke & Imhof, 2003 ▸). 
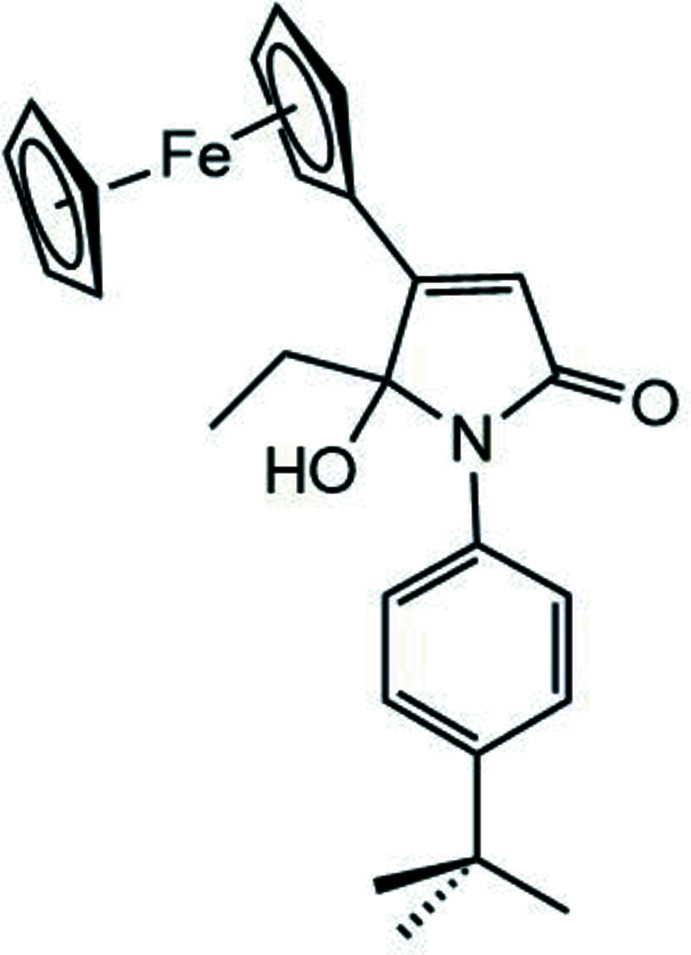


## 2. Structural commentary

**Figure 1:**
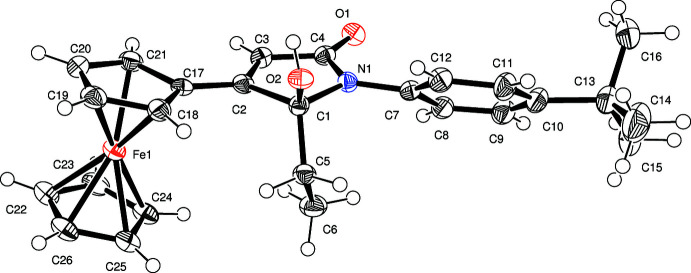
Mol­ecular structure of the *S*-enanti­omer of the title compound showing the numbering scheme. Non-hydrogen atoms are drawn as displacement ellipsoids at the 50% probability level.

The title compound, *rac*-1-(4-*tert-*butyl­phen­yl)-5-ethyl-4-ferrocenyl-5-hydroxyl-1*H*-pyrrol-2(5*H*)-one, C_26_H_29_FeNO_2_, is derived from 1-(4-*tert*-butyl­phen­yl)-2-ethyl-3-ferrocenyl­pyrrole by an oxidation reaction. Therefore, a new centre of chirality is created at C1, which used to be an *sp* ^2^ carbon atom in the starting compound. Since no chiral reaction conditions were applied, a racemate of the title compound is produced. The title compound also crystallizes as a racemic mixture in the centrosymmetric space group *P*2_1_/*n*. The mol­ecular structure of the *S*-enanti­omer is shown in Fig. 1 ▸. The central heterocyclic ring system N1/C1–C4 shows torsional angles of 13.7 (2)° with respect to the attached cyclo­penta­dienyl ring and of 43.6 (7)° with the major component of the disordered phenyl group bound to N1. The 4-*tert*-butyl­phenyl group, as well as the non-substituted Cp ring, are disordered with s.o.f. values of 0.589 (16) and 0.411 (16). Bond lengths and angles are of expected values with the C2—C3 bond length of 1.336 (5) Å, clearly indicating a double bond. In addition, the N1—C4 bond [1.366 (5) Å] is shortened with respect to the other nitro­gen carbon bonds, as is typical for amides.

## 3. Supra­molecular features

**Figure 2:**
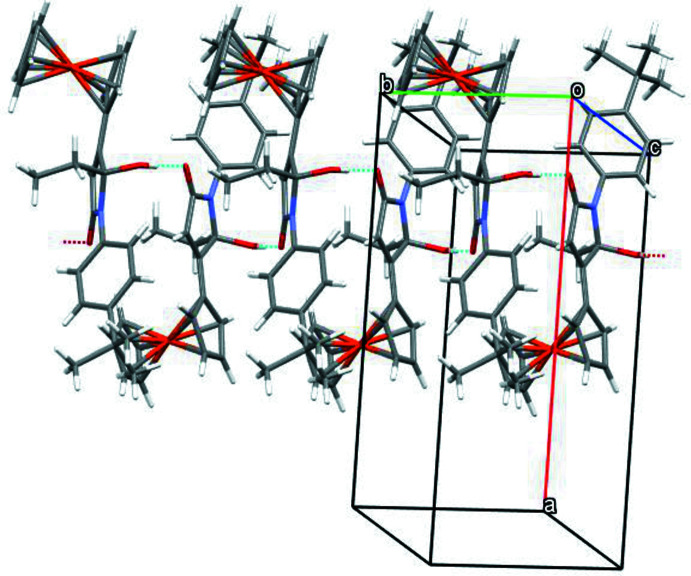
Infinite chain of the *S*-enanti­omers along the *b*-axis.

In the crystal, mol­ecules with the same absolute configuration at C1 are linked into infinite chains along the *b*-axis direction by O—H⋯O hydrogen bonds of the *C*(6) type (Bernstein *et al.*, 1995 ▸) between the hy­droxy substituent and the carbonyl oxygen atom of an adjacent mol­ecule (Fig. 2 ▸, Table 1 ▸). In addition, there are weak contacts between carbon atoms of the phenyl ring and H3*A* and H23*A*.

## 4. Database survey

Some years ago, we published the crystal structure of a derivative of the title compound, *N*-methyl-5-ethyl-5-hy­droxy-4-phenyl-1*H*-pyrrol-2(5*H*)-one CSD (Groom *et al.*, 2016 ▸) refcode ULUJUG; Dönnecke & Imhof, 2003 ▸]. The compound shows almost identical structural features concerning the pyrrolone ring system and also crystallizes as a racemate in the space group *Pna*2_1_.

Compounds with related heterocyclic systems such as ferrocenyl-substituted male­imides or a 1,5-di­hydro-2*H*-pyrrole-2-one with an imino substituent at C5 have also been reported (CATTOI: Mathur *et al.*, 2012 ▸; TASNEI, TASNIM: Hildebrandt *et al.*, 2012 ▸; ZEPLOY, ZEPLUE, ZEPMAL: Jha *et al.*, 2017 ▸; CIVCUI: Raghuvanshi *et al.*, 2017 ▸).

## 5. Synthesis and crystallization

0.5 mmol (200 mg) of 1-(4-*tert*-butyl­phen­yl)-2-ethyl-3-ferrocenyl­pyrrole were treated with 5 mol% *p*-toluene sulfonic acid and were dissolved in 1.0 mL of anhydrous ethanol. The solution was placed in a 10 mL screw-cap vessel closed with parafilm. The process of the oxidation reaction was followed by thin layer chromatography and it could be observed that the reaction was finished after approximately 8 days. The reaction mixture was transferred to a Schlenk tube, the solvent was removed *in vacuo* and the remaining oily residue was purified by column chromatography (10 × 2 cm, silica) using CH_2_Cl_2_ as the eluent. Slow evaporation of the solvent at ambient temperature led to the formation of crystalline material of the title compound (yield 183 mg, 83%). ^1^H NMR (400 MHz, CDCl_3_, 298 K): (ppm) = 0.55 (*t*, 3H, *J*_HH_ = 7.4 Hz, CH_3_); 1.31 (*s*, 9H, CH_3_); 1.92 (*q*, 2H, *J*_HH_ = 7.5 Hz, CH_2_); 2.84 (*s*, 1H, OH); 4.17 (*s*, 5H, Cp); 4.44–4.50 (*m*, 2H, Cp*R*); 4.72–4.73 (*m*, 2H, Cp*R*); 6.24 (*s*, 1H, =CH); 7.37–7.43 (*m*, 2H, CH_Ph_); 7.48–7.52 (*m*, 2H, CH_Ph_). ^13^C NMR (100 MHz, CDCl_3_, 298 K): (ppm) = 7.80 (CH_3_); 26.37 (CH_2_); 31.32 (CH_3_); 34.50 (C); 68.03 (Cp*R*); 68.85 (Cp*R*); 70.03 (Cp); 72.96 (Cp*R*); 95.55 (C); 118.48 (=CH); 125.44 (CH_Ph_); 125.86 (CH_Ph_); 135.19 (C_Ph_); 149.21 (C_Ph_); 160.63 (C); 169.10 (C=O). MS (DEI): *m*/*z* (%) = 443 (96) [*M* ^+^]; 427 (76) [*M* ^+^ − O]; 426 (40) [*M* ^+^ − OH]; 425 (75) [*M* ^+^ − H_2_O]; 398 (22) [*M* ^+^ − 3CH_3_]; 360 (98) [*M* ^+^ − C_5_H_5_ − H_2_O]; 322 (48) [*M* ^+^ − C_5_H_5_Fe]; 305 (58) [*M* ^+^ − C_5_H_5_Fe − OH]; 294 (64) [*M* ^+^ − C_5_H_5_Fe − CO].

## 6. Refinement

Crystal data, data collection and structure refinement details are summarized in Table 2 ▸. The hydrogen atom of the hy­droxy substituent (H1*O*2) was located in a difference-Fourier map and refined freely. All carbon-bound hydrogen atoms were placed in idealized positions and refined using a riding model with isotropic displacement parameters *U*_iso_(H) = 1.2*U_eq_* (C) for methyl­ene and aromatic hydrogen atoms and H3 and *U*_iso_(H) = 1.5*U_eq_* (C) for methyl groups. The *p*-^*t*^BuC_6_H_4_ and Cp groups are disordered over two positions and were found to refine well with only one free variable. The proportion of the two positions is 58.94:41.06%. SIMU, RIGU, SAME, SADI and FLAT instructions were used to restrain the geometry and displacement parameters of the disordered moieties.

## Supplementary Material

Crystal structure: contains datablock(s) I. DOI: 10.1107/S2056989023001858/jq2026sup1.cif

Structure factors: contains datablock(s) I. DOI: 10.1107/S2056989023001858/jq2026Isup2.hkl

CCDC reference: 961575

Additional supporting information:  crystallographic information; 3D view; checkCIF report

## Figures and Tables

**Table 1:** Hydrogen-bond geometry (Å, °)

*D*—H⋯*A*	*D*—H	H⋯*A*	*D*⋯*A*	*D*—H⋯*A*
O2—H1*O*2⋯O1^i^	0.80 (4)	1.91 (5)	2.699 (4)	166 (5)

Symmetry code: (i) 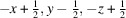 .

**Table 2:** Experimental details

Crystal data	
Chemical formula	[Fe(C_5_H_5_)(C_21_H_24_NO_2_)]
*M*_r_	443.35
Crystal system, space group	Monoclinic, *P*2_1_/*n*
Temperature (K)	133
*a*, *b*, *c* (Å)	15.7256 (5), 7.0155 (2), 20.0725 (6)
β (°)	101.242 (2)
*V* (Å^3^)	2171.97 (11)
*Z*	4
Radiation type	Mo *K*α
μ (mm^−1^)	0.72
Crystal size (mm)	0.09 × 0.07 × 0.05

Data collection	
Diffractometer	Nonius KappaCCD
Absorption correction	Multi-scan (*SADABS*; Krause *et al.*, 2015 ▸)
*T*_min_, *T*_max_	0.693, 0.746
No. of measured, independent and observed [*I* > 2σ(*I*)] reflections	12993, 4945, 3348
*R*_int_	0.083
(sin θ/λ)_max_ (Å^−1^)	0.649

Refinement	
*R*[*F* ^2^ > 2σ(*F* ^2^)], *wR*(*F* ^2^), *S*	0.073, 0.144, 1.15
No. of reflections	4945
No. of parameters	418
No. of restraints	950
H-atom treatment	H atoms treated by a mixture of independent and constrained refinement
Δρ_max_, Δρ_min_ (e Å^−3^)	0.66, −0.46

Computer programs: *COLLECT* (Nonius 1998 ▸), *DENZO* (Otwinowski & Minor, 1997 ▸), *SHELXS97* (Sheldrick, 2008 ▸), *SHELXL* (Sheldrick, 2015 ▸) and *ORTEP-3 for Windows* (Farrugia, 2012 ▸).

## supplementary crystallographic information

### Crystal data

**Table:**

[Fe(C_5_H_5_)(C_21_H_24_NO_2_)]	*F*(000) = 936
*M**_r_* = 443.35	*D*_x_ = 1.356 Mg m^−^^3^
Monoclinic, *P*2_1_/*n*	Mo *K*α radiation, λ = 0.71073 Å
*a* = 15.7256 (5) Å	Cell parameters from 12993 reflections
*b* = 7.0155 (2) Å	θ = 2.6–27.5°
*c* = 20.0725 (6) Å	µ = 0.72 mm^−^^1^
β = 101.242 (2)°	*T* = 133 K
*V* = 2171.97 (11) Å^3^	Prism, red-brown
*Z* = 4	0.09 × 0.07 × 0.05 mm

### Data collection

**Table:**

Nonius KappaCCD diffractometer	3348 reflections with *I* > 2σ(*I*)
phi + ω – scans	*R*_int_ = 0.083
Absorption correction: multi-scan (SADABS; Krause *et al.*, 2015)	θ_max_ = 27.5°, θ_min_ = 2.6°
*T*_min_ = 0.693, *T*_max_ = 0.746	*h* = −20→20
12993 measured reflections	*k* = −8→9
4945 independent reflections	*l* = −26→22

### Refinement

**Table:**

Refinement on *F*^2^	Primary atom site location: structure-invariant direct methods
Least-squares matrix: full	Secondary atom site location: difference Fourier map
*R*[*F*^2^ > 2σ(*F*^2^)] = 0.073	Hydrogen site location: mixed
*wR*(*F*^2^) = 0.144	H atoms treated by a mixture of independent and constrained refinement
*S* = 1.15	*w* = 1/[σ^2^(*F*_o_^2^) + (0.0092*P*)^2^ + 6.8638*P*] where *P* = (*F*_o_^2^ + 2*F*_c_^2^)/3
4945 reflections	(Δ/σ)_max_ = 0.001
418 parameters	Δρ_max_ = 0.66 e Å^−^^3^
950 restraints	Δρ_min_ = −0.46 e Å^−^^3^

### Special details

**Table:**

Geometry. All esds (except the esd in the dihedral angle between two l.s. planes) are estimated using the full covariance matrix. The cell esds are taken into account individually in the estimation of esds in distances, angles and torsion angles; correlations between esds in cell parameters are only used when they are defined by crystal symmetry. An approximate (isotropic) treatment of cell esds is used for estimating esds involving l.s. planes.

### Fractional atomic coordinates and isotropic or equivalent isotropic displacement parameters (Å^2^)

**Table:**

	*x*	*y*	*z*	*U*_iso_*/*U*_eq_	Occ. (<1)
Fe1	0.56011 (4)	1.17446 (9)	0.36865 (3)	0.02460 (17)	
O1	0.15960 (18)	1.0828 (4)	0.21878 (14)	0.0262 (7)	
O2	0.32476 (19)	0.7788 (4)	0.39389 (14)	0.0234 (6)	
H1O2	0.327 (3)	0.705 (7)	0.364 (2)	0.025 (13)*	
N1	0.2200 (2)	0.9892 (5)	0.32817 (15)	0.0209 (7)	
C1	0.3081 (3)	0.9674 (6)	0.37048 (19)	0.0211 (8)	
C2	0.3651 (3)	1.0215 (5)	0.31929 (19)	0.0215 (8)	
C3	0.3144 (3)	1.0722 (5)	0.2606 (2)	0.0221 (9)	
H3A	0.334929	1.115373	0.221737	0.027*	
C4	0.2233 (3)	1.0520 (5)	0.2643 (2)	0.0222 (8)	
C5	0.3187 (3)	1.0948 (6)	0.4330 (2)	0.0249 (9)	
H5A	0.380511	1.095715	0.455599	0.030*	
H5B	0.285216	1.039071	0.465217	0.030*	
C6	0.2889 (3)	1.2999 (6)	0.4179 (2)	0.0356 (11)	
H6A	0.308703	1.378829	0.458206	0.053*	
H6B	0.225458	1.303725	0.405795	0.053*	
H6C	0.313400	1.348858	0.379969	0.053*	
C7	0.1414 (9)	0.960 (4)	0.3517 (13)	0.0260 (17)	0.589 (16)
C8	0.0759 (10)	1.095 (3)	0.3342 (10)	0.030 (2)	0.589 (16)
H8A	0.085728	1.206923	0.310245	0.036*	0.589 (16)
C9	−0.0036 (9)	1.0638 (19)	0.3521 (8)	0.036 (2)	0.589 (16)
H9A	−0.048051	1.156377	0.339971	0.043*	0.589 (16)
C10	−0.0208 (7)	0.9016 (19)	0.3873 (8)	0.0370 (19)	0.589 (16)
C11	0.0469 (9)	0.776 (2)	0.4080 (11)	0.035 (2)	0.589 (16)
H11A	0.038755	0.669994	0.435654	0.042*	0.589 (16)
C12	0.1271 (9)	0.801 (4)	0.3891 (14)	0.0320 (18)	0.589 (16)
H12A	0.171823	0.709245	0.401815	0.038*	0.589 (16)
C13	−0.1109 (7)	0.8565 (17)	0.4023 (6)	0.046 (2)	0.589 (16)
C14	−0.1043 (8)	0.753 (2)	0.4702 (6)	0.067 (3)	0.589 (16)
H14A	−0.162596	0.725677	0.478082	0.101*	0.589 (16)
H14B	−0.073419	0.833891	0.506880	0.101*	0.589 (16)
H14C	−0.072478	0.633291	0.469072	0.101*	0.589 (16)
C15	−0.1633 (8)	1.0388 (17)	0.4038 (8)	0.060 (3)	0.589 (16)
H15A	−0.173123	1.099364	0.359002	0.090*	0.589 (16)
H15B	−0.131301	1.126349	0.437742	0.090*	0.589 (16)
H15C	−0.219260	1.007828	0.415800	0.090*	0.589 (16)
C16	−0.1578 (13)	0.726 (3)	0.3446 (8)	0.059 (4)	0.589 (16)
H16A	−0.155996	0.785115	0.300598	0.089*	0.589 (16)
H16B	−0.218311	0.709334	0.349065	0.089*	0.589 (16)
H16C	−0.128916	0.602035	0.347502	0.089*	0.589 (16)
C7A	0.1408 (13)	0.953 (5)	0.3512 (18)	0.027 (2)	0.411 (16)
C8A	0.0670 (15)	1.065 (4)	0.3316 (14)	0.031 (2)	0.411 (16)
H8B	0.070014	1.175646	0.305131	0.037*	0.411 (16)
C9A	−0.0109 (13)	1.017 (3)	0.3500 (11)	0.035 (2)	0.411 (16)
H9B	−0.060483	1.094123	0.335113	0.042*	0.411 (16)
C10A	−0.0176 (11)	0.857 (3)	0.3901 (12)	0.037 (2)	0.411 (16)
C11A	0.0559 (12)	0.745 (4)	0.4084 (16)	0.034 (2)	0.411 (16)
H11B	0.051858	0.629669	0.432550	0.041*	0.411 (16)
C12A	0.1357 (13)	0.795 (5)	0.393 (2)	0.030 (2)	0.411 (16)
H12B	0.186103	0.722100	0.409741	0.036*	0.411 (16)
C13A	−0.1078 (10)	0.806 (2)	0.4038 (8)	0.047 (2)	0.411 (16)
C14A	−0.1049 (11)	0.639 (3)	0.4527 (9)	0.064 (4)	0.411 (16)
H14D	−0.089734	0.521865	0.430827	0.096*	0.411 (16)
H14E	−0.161791	0.622925	0.465023	0.096*	0.411 (16)
H14F	−0.061165	0.663229	0.493786	0.096*	0.411 (16)
C15A	−0.1447 (12)	0.976 (3)	0.4372 (10)	0.060 (4)	0.411 (16)
H15D	−0.111442	0.993369	0.483436	0.090*	0.411 (16)
H15E	−0.205564	0.951393	0.438885	0.090*	0.411 (16)
H15F	−0.140515	1.091388	0.410557	0.090*	0.411 (16)
C16A	−0.1690 (18)	0.764 (4)	0.3356 (10)	0.054 (4)	0.411 (16)
H16D	−0.149443	0.649419	0.315222	0.082*	0.411 (16)
H16E	−0.168575	0.872598	0.304820	0.082*	0.411 (16)
H16F	−0.228044	0.744424	0.343292	0.082*	0.411 (16)
C17	0.4581 (3)	0.9933 (5)	0.3329 (2)	0.0223 (8)	
C18	0.5120 (3)	0.9167 (6)	0.3926 (2)	0.0260 (9)	
H18A	0.493651	0.885965	0.433676	0.031*	
C19	0.5973 (3)	0.8941 (6)	0.3805 (2)	0.0296 (10)	
H19A	0.645737	0.845755	0.411777	0.035*	
C20	0.5972 (3)	0.9570 (6)	0.3133 (2)	0.0285 (10)	
H20A	0.645796	0.957145	0.291622	0.034*	
C21	0.5132 (3)	1.0190 (6)	0.2841 (2)	0.0278 (9)	
H21A	0.495721	1.069417	0.239598	0.033*	
C22	0.6534 (9)	1.379 (2)	0.3699 (7)	0.032 (3)	0.589 (16)
H22A	0.703759	1.368664	0.350567	0.038*	0.589 (16)
C23	0.5716 (9)	1.446 (2)	0.3365 (6)	0.032 (3)	0.589 (16)
H23A	0.557045	1.488513	0.290781	0.038*	0.589 (16)
C24	0.5144 (8)	1.439 (2)	0.3840 (7)	0.030 (3)	0.589 (16)
H24A	0.455170	1.476006	0.375409	0.036*	0.589 (16)
C25	0.5618 (10)	1.366 (3)	0.4457 (7)	0.033 (3)	0.589 (16)
H25A	0.540087	1.346345	0.486172	0.039*	0.589 (16)
C26	0.6475 (9)	1.329 (3)	0.4369 (7)	0.030 (3)	0.589 (16)
H26A	0.693122	1.278396	0.470414	0.036*	0.589 (16)
C22A	0.6328 (12)	1.405 (3)	0.3552 (10)	0.032 (4)	0.411 (16)
H22B	0.670190	1.410304	0.323393	0.038*	0.411 (16)
C23A	0.5445 (12)	1.458 (3)	0.3431 (9)	0.028 (4)	0.411 (16)
H23B	0.512555	1.507323	0.301608	0.034*	0.411 (16)
C24A	0.5115 (12)	1.425 (3)	0.4027 (10)	0.028 (4)	0.411 (16)
H24B	0.453618	1.446702	0.408183	0.034*	0.411 (16)
C25A	0.5806 (15)	1.354 (4)	0.4528 (10)	0.030 (4)	0.411 (16)
H25B	0.577286	1.320987	0.498091	0.036*	0.411 (16)
C26A	0.6554 (13)	1.342 (4)	0.4233 (11)	0.034 (5)	0.411 (16)
H26B	0.711089	1.299459	0.445504	0.041*	0.411 (16)

### Atomic displacement parameters (Å^2^)

**Table:**

	*U*^11^	*U*^22^	*U*^33^	*U*^12^	*U*^13^	*U*^23^
Fe1	0.0254 (3)	0.0215 (3)	0.0259 (3)	−0.0029 (3)	0.0025 (2)	−0.0012 (3)
O1	0.0284 (16)	0.0264 (16)	0.0220 (14)	0.0004 (13)	0.0004 (12)	0.0042 (12)
O2	0.0298 (16)	0.0207 (15)	0.0187 (14)	0.0008 (12)	0.0021 (12)	0.0002 (12)
N1	0.0201 (17)	0.0241 (18)	0.0181 (16)	−0.0013 (14)	0.0024 (13)	0.0002 (14)
C1	0.022 (2)	0.022 (2)	0.0202 (19)	−0.0008 (16)	0.0061 (16)	0.0003 (17)
C2	0.027 (2)	0.0162 (19)	0.023 (2)	−0.0048 (16)	0.0068 (17)	−0.0007 (16)
C3	0.029 (2)	0.018 (2)	0.0205 (19)	−0.0003 (17)	0.0079 (17)	0.0041 (16)
C4	0.027 (2)	0.0181 (19)	0.023 (2)	−0.0002 (16)	0.0066 (17)	0.0001 (17)
C5	0.028 (2)	0.026 (2)	0.021 (2)	−0.0026 (18)	0.0048 (17)	−0.0021 (17)
C6	0.043 (3)	0.028 (2)	0.037 (3)	−0.004 (2)	0.010 (2)	−0.008 (2)
C7	0.024 (3)	0.034 (4)	0.020 (3)	−0.003 (3)	0.006 (3)	−0.007 (3)
C8	0.023 (4)	0.042 (5)	0.024 (3)	0.001 (3)	0.002 (3)	−0.006 (4)
C9	0.027 (3)	0.048 (5)	0.033 (3)	0.002 (3)	0.006 (3)	−0.008 (4)
C10	0.028 (3)	0.055 (5)	0.031 (3)	−0.004 (3)	0.011 (3)	−0.010 (4)
C11	0.033 (3)	0.049 (5)	0.026 (3)	−0.006 (3)	0.013 (3)	0.001 (4)
C12	0.032 (3)	0.039 (3)	0.027 (4)	−0.004 (3)	0.008 (3)	−0.003 (3)
C13	0.032 (3)	0.068 (5)	0.043 (3)	−0.010 (3)	0.017 (3)	−0.014 (4)
C14	0.058 (6)	0.094 (8)	0.057 (5)	−0.012 (6)	0.032 (5)	0.002 (5)
C15	0.033 (5)	0.075 (6)	0.078 (7)	−0.011 (4)	0.026 (6)	−0.019 (6)
C16	0.040 (7)	0.083 (8)	0.060 (6)	−0.024 (6)	0.021 (5)	−0.023 (6)
C7A	0.025 (4)	0.037 (4)	0.020 (4)	−0.003 (3)	0.005 (3)	−0.007 (4)
C8A	0.027 (4)	0.042 (5)	0.025 (4)	0.000 (4)	0.004 (4)	−0.006 (4)
C9A	0.026 (4)	0.048 (6)	0.031 (4)	0.001 (4)	0.004 (3)	−0.009 (4)
C10A	0.029 (3)	0.053 (5)	0.030 (3)	−0.006 (3)	0.011 (3)	−0.008 (4)
C11A	0.031 (4)	0.046 (5)	0.028 (4)	−0.006 (4)	0.011 (4)	−0.005 (4)
C12A	0.029 (4)	0.041 (4)	0.022 (4)	−0.005 (4)	0.008 (4)	−0.003 (3)
C13A	0.032 (4)	0.070 (5)	0.044 (4)	−0.010 (4)	0.017 (3)	−0.009 (4)
C14A	0.043 (7)	0.089 (9)	0.066 (8)	−0.019 (7)	0.023 (6)	0.010 (7)
C15A	0.040 (7)	0.083 (8)	0.063 (8)	−0.014 (6)	0.026 (6)	−0.027 (7)
C16A	0.040 (7)	0.069 (9)	0.054 (7)	−0.006 (7)	0.010 (6)	−0.019 (7)
C17	0.025 (2)	0.0170 (19)	0.024 (2)	−0.0027 (16)	0.0038 (17)	−0.0063 (17)
C18	0.028 (2)	0.022 (2)	0.029 (2)	−0.0047 (18)	0.0077 (18)	−0.0020 (18)
C19	0.031 (2)	0.020 (2)	0.036 (2)	−0.0020 (18)	0.003 (2)	−0.0037 (19)
C20	0.025 (2)	0.031 (2)	0.030 (2)	−0.0003 (18)	0.0077 (18)	−0.008 (2)
C21	0.030 (2)	0.029 (2)	0.023 (2)	−0.0045 (19)	0.0033 (18)	−0.0045 (19)
C22	0.030 (5)	0.030 (6)	0.033 (6)	−0.006 (4)	0.000 (4)	−0.001 (4)
C23	0.040 (7)	0.021 (5)	0.032 (4)	−0.008 (5)	0.001 (4)	0.002 (4)
C24	0.032 (5)	0.022 (6)	0.032 (6)	−0.005 (4)	−0.002 (4)	−0.002 (5)
C25	0.031 (6)	0.033 (7)	0.033 (5)	−0.006 (5)	0.001 (4)	−0.010 (5)
C26	0.033 (5)	0.023 (5)	0.031 (5)	−0.008 (4)	−0.004 (4)	0.002 (5)
C22A	0.027 (8)	0.032 (8)	0.037 (8)	−0.012 (6)	0.004 (6)	−0.004 (6)
C23A	0.033 (8)	0.014 (6)	0.038 (7)	−0.004 (6)	0.005 (5)	0.007 (5)
C24A	0.033 (6)	0.016 (7)	0.035 (8)	−0.007 (5)	0.005 (6)	0.006 (6)
C25A	0.038 (9)	0.022 (7)	0.027 (6)	0.004 (7)	−0.003 (5)	0.000 (6)
C26A	0.030 (6)	0.035 (10)	0.034 (8)	−0.004 (6)	−0.002 (5)	−0.013 (7)

### Geometric parameters (Å, º)

**Table:**

Fe1—C22A	2.03 (2)	C16—H16C	0.9800
Fe1—C23	2.030 (14)	C7A—C8A	1.392 (12)
Fe1—C21	2.031 (4)	C7A—C12A	1.395 (12)
Fe1—C24	2.034 (14)	C8A—C9A	1.388 (12)
Fe1—C20	2.039 (4)	C8A—H8B	0.9500
Fe1—C22	2.047 (15)	C9A—C10A	1.396 (12)
Fe1—C25	2.05 (2)	C9A—H9B	0.9500
Fe1—C26A	2.05 (3)	C10A—C11A	1.387 (11)
Fe1—C26	2.050 (19)	C10A—C13A	1.539 (12)
Fe1—C19	2.052 (4)	C11A—C12A	1.399 (12)
Fe1—C18	2.053 (4)	C11A—H11B	0.9500
Fe1—C23A	2.056 (19)	C12A—H12B	0.9500
O1—C4	1.236 (5)	C13A—C14A	1.525 (12)
O2—C1	1.411 (5)	C13A—C15A	1.536 (13)
O2—H1O2	0.80 (4)	C13A—C16A	1.541 (13)
N1—C4	1.366 (5)	C14A—H14D	0.9800
N1—C7	1.422 (9)	C14A—H14E	0.9800
N1—C7A	1.433 (12)	C14A—H14F	0.9800
N1—C1	1.485 (5)	C15A—H15D	0.9800
C1—C5	1.523 (5)	C15A—H15E	0.9800
C1—C2	1.537 (5)	C15A—H15F	0.9800
C2—C3	1.336 (5)	C16A—H16D	0.9800
C2—C17	1.447 (5)	C16A—H16E	0.9800
C3—C4	1.455 (6)	C16A—H16F	0.9800
C3—H3A	0.9500	C17—C18	1.431 (6)
C5—C6	1.525 (6)	C17—C21	1.441 (6)
C5—H5A	0.9900	C18—C19	1.417 (6)
C5—H5B	0.9900	C18—H18A	0.9500
C6—H6A	0.9800	C19—C20	1.420 (6)
C6—H6B	0.9800	C19—H19A	0.9500
C6—H6C	0.9800	C20—C21	1.405 (6)
C7—C12	1.389 (10)	C20—H20A	0.9500
C7—C8	1.390 (10)	C21—H21A	0.9500
C8—C9	1.385 (10)	C22—C26	1.411 (10)
C8—H8A	0.9500	C22—C23	1.411 (10)
C9—C10	1.393 (10)	C22—H22A	0.9500
C9—H9A	0.9500	C23—C24	1.433 (10)
C10—C11	1.384 (9)	C23—H23A	0.9500
C10—C13	1.539 (10)	C24—C25	1.411 (10)
C11—C12	1.397 (10)	C24—H24A	0.9500
C11—H11A	0.9500	C25—C26	1.418 (10)
C12—H12A	0.9500	C25—H25A	0.9500
C13—C15	1.525 (10)	C26—H26A	0.9500
C13—C14	1.530 (11)	C22A—C23A	1.411 (12)
C13—C16	1.544 (11)	C22A—C26A	1.414 (12)
C14—H14A	0.9800	C22A—H22B	0.9500
C14—H14B	0.9800	C23A—C24A	1.413 (12)
C14—H14C	0.9800	C23A—H23B	0.9500
C15—H15A	0.9800	C24A—C25A	1.419 (12)
C15—H15B	0.9800	C24A—H24B	0.9500
C15—H15C	0.9800	C25A—C26A	1.419 (12)
C16—H16A	0.9800	C25A—H25B	0.9500
C16—H16B	0.9800	C26A—H26B	0.9500
			
C22A—Fe1—C21	116.1 (6)	C9A—C8A—C7A	121.3 (13)
C23—Fe1—C21	106.2 (4)	C9A—C8A—H8B	119.4
C23—Fe1—C24	41.3 (4)	C7A—C8A—H8B	119.4
C21—Fe1—C24	122.3 (4)	C8A—C9A—C10A	121.2 (12)
C22A—Fe1—C20	107.2 (5)	C8A—C9A—H9B	119.4
C23—Fe1—C20	118.4 (4)	C10A—C9A—H9B	119.4
C21—Fe1—C20	40.40 (17)	C11A—C10A—C9A	117.1 (11)
C24—Fe1—C20	155.8 (4)	C11A—C10A—C13A	124.7 (11)
C23—Fe1—C22	40.5 (3)	C9A—C10A—C13A	117.8 (11)
C21—Fe1—C22	121.9 (4)	C10A—C11A—C12A	122.3 (13)
C24—Fe1—C22	68.5 (4)	C10A—C11A—H11B	118.8
C20—Fe1—C22	104.5 (4)	C12A—C11A—H11B	118.8
C23—Fe1—C25	68.6 (5)	C7A—C12A—C11A	119.6 (13)
C21—Fe1—C25	159.0 (4)	C7A—C12A—H12B	120.2
C24—Fe1—C25	40.4 (4)	C11A—C12A—H12B	120.2
C20—Fe1—C25	160.3 (4)	C14A—C13A—C15A	106.5 (12)
C22—Fe1—C25	68.1 (5)	C14A—C13A—C10A	112.4 (11)
C22A—Fe1—C26A	40.6 (5)	C15A—C13A—C10A	109.7 (11)
C21—Fe1—C26A	149.9 (6)	C14A—C13A—C16A	111.2 (13)
C20—Fe1—C26A	117.7 (7)	C15A—C13A—C16A	108.1 (13)
C23—Fe1—C26	68.1 (5)	C10A—C13A—C16A	108.9 (14)
C21—Fe1—C26	158.2 (4)	C13A—C14A—H14D	109.5
C24—Fe1—C26	68.2 (5)	C13A—C14A—H14E	109.5
C20—Fe1—C26	122.2 (5)	H14D—C14A—H14E	109.5
C22—Fe1—C26	40.3 (3)	C13A—C14A—H14F	109.5
C25—Fe1—C26	40.5 (4)	H14D—C14A—H14F	109.5
C22A—Fe1—C19	128.6 (6)	H14E—C14A—H14F	109.5
C23—Fe1—C19	153.5 (4)	C13A—C15A—H15D	109.5
C21—Fe1—C19	68.37 (18)	C13A—C15A—H15E	109.5
C24—Fe1—C19	163.2 (4)	H15D—C15A—H15E	109.5
C20—Fe1—C19	40.62 (17)	C13A—C15A—H15F	109.5
C22—Fe1—C19	118.8 (4)	H15D—C15A—H15F	109.5
C25—Fe1—C19	125.5 (5)	H15E—C15A—H15F	109.5
C26A—Fe1—C19	109.0 (7)	C13A—C16A—H16D	109.5
C26—Fe1—C19	106.8 (5)	C13A—C16A—H16E	109.5
C22A—Fe1—C18	167.5 (6)	H16D—C16A—H16E	109.5
C23—Fe1—C18	163.8 (4)	C13A—C16A—H16F	109.5
C21—Fe1—C18	68.44 (17)	H16D—C16A—H16F	109.5
C24—Fe1—C18	127.6 (4)	H16E—C16A—H16F	109.5
C20—Fe1—C18	68.05 (18)	C18—C17—C21	106.2 (4)
C22—Fe1—C18	155.4 (4)	C18—C17—C2	128.5 (4)
C25—Fe1—C18	110.7 (5)	C21—C17—C2	125.0 (4)
C26A—Fe1—C18	130.2 (6)	C18—C17—Fe1	69.3 (2)
C26—Fe1—C18	122.5 (4)	C21—C17—Fe1	68.2 (2)
C19—Fe1—C18	40.39 (17)	C2—C17—Fe1	132.0 (3)
C22A—Fe1—C23A	40.4 (4)	C19—C18—C17	109.0 (4)
C21—Fe1—C23A	107.7 (5)	C19—C18—Fe1	69.8 (2)
C20—Fe1—C23A	128.3 (6)	C17—C18—Fe1	70.0 (2)
C26A—Fe1—C23A	67.5 (7)	C19—C18—H18A	125.5
C19—Fe1—C23A	166.7 (6)	C17—C18—H18A	125.5
C18—Fe1—C23A	151.5 (5)	Fe1—C18—H18A	126.3
C1—O2—H1O2	113 (3)	C18—C19—C20	107.6 (4)
C4—N1—C7	123.5 (10)	C18—C19—Fe1	69.8 (2)
C4—N1—C7A	123.6 (14)	C20—C19—Fe1	69.2 (2)
C4—N1—C1	111.6 (3)	C18—C19—H19A	126.2
C7—N1—C1	124.8 (10)	C20—C19—H19A	126.2
C7A—N1—C1	124.7 (14)	Fe1—C19—H19A	126.4
O2—C1—N1	112.2 (3)	C21—C20—C19	108.6 (4)
O2—C1—C5	107.0 (3)	C21—C20—Fe1	69.5 (2)
N1—C1—C5	110.5 (3)	C19—C20—Fe1	70.2 (2)
O2—C1—C2	111.1 (3)	C21—C20—H20A	125.7
N1—C1—C2	101.1 (3)	C19—C20—H20A	125.7
C5—C1—C2	115.0 (3)	Fe1—C20—H20A	126.2
C3—C2—C17	127.6 (4)	C20—C21—C17	108.6 (4)
C3—C2—C1	109.3 (4)	C20—C21—Fe1	70.1 (2)
C17—C2—C1	122.6 (3)	C17—C21—Fe1	70.6 (2)
C2—C3—C4	110.7 (4)	C20—C21—H21A	125.7
C2—C3—H3A	124.6	C17—C21—H21A	125.7
C4—C3—H3A	124.6	Fe1—C21—H21A	125.2
O1—C4—N1	125.2 (4)	C26—C22—C23	108.2 (9)
O1—C4—C3	127.5 (4)	C26—C22—Fe1	70.0 (9)
N1—C4—C3	107.3 (3)	C23—C22—Fe1	69.1 (7)
C1—C5—C6	114.2 (3)	C26—C22—H22A	125.9
C1—C5—H5A	108.7	C23—C22—H22A	125.9
C6—C5—H5A	108.7	Fe1—C22—H22A	126.6
C1—C5—H5B	108.7	C22—C23—C24	107.7 (8)
C6—C5—H5B	108.7	C22—C23—Fe1	70.4 (7)
H5A—C5—H5B	107.6	C24—C23—Fe1	69.5 (6)
C5—C6—H6A	109.5	C22—C23—H23A	126.1
C5—C6—H6B	109.5	C24—C23—H23A	126.1
H6A—C6—H6B	109.5	Fe1—C23—H23A	125.6
C5—C6—H6C	109.5	C25—C24—C23	107.7 (9)
H6A—C6—H6C	109.5	C25—C24—Fe1	70.3 (10)
H6B—C6—H6C	109.5	C23—C24—Fe1	69.2 (7)
C12—C7—C8	119.6 (9)	C25—C24—H24A	126.1
C12—C7—N1	122.5 (14)	C23—C24—H24A	126.1
C8—C7—N1	117.9 (14)	Fe1—C24—H24A	125.9
C9—C8—C7	119.3 (9)	C24—C25—C26	108.1 (9)
C9—C8—H8A	120.3	C24—C25—Fe1	69.3 (9)
C7—C8—H8A	120.3	C26—C25—Fe1	69.9 (10)
C8—C9—C10	122.3 (9)	C24—C25—H25A	126.0
C8—C9—H9A	118.9	C26—C25—H25A	126.0
C10—C9—H9A	118.9	Fe1—C25—H25A	126.5
C11—C10—C9	117.4 (8)	C22—C26—C25	108.3 (9)
C11—C10—C13	119.7 (9)	C22—C26—Fe1	69.7 (9)
C9—C10—C13	122.9 (8)	C25—C26—Fe1	69.6 (10)
C10—C11—C12	121.4 (10)	C22—C26—H26A	125.9
C10—C11—H11A	119.3	C25—C26—H26A	125.9
C12—C11—H11A	119.3	Fe1—C26—H26A	126.3
C7—C12—C11	119.9 (10)	C23A—C22A—C26A	107.6 (11)
C7—C12—H12A	120.1	C23A—C22A—Fe1	70.9 (10)
C11—C12—H12A	120.1	C26A—C22A—Fe1	70.5 (14)
C15—C13—C14	108.8 (9)	C23A—C22A—H22B	126.2
C15—C13—C10	110.7 (8)	C26A—C22A—H22B	126.2
C14—C13—C10	111.4 (8)	Fe1—C22A—H22B	124.1
C15—C13—C16	109.3 (10)	C22A—C23A—C24A	108.8 (11)
C14—C13—C16	109.1 (10)	C22A—C23A—Fe1	68.7 (10)
C10—C13—C16	107.4 (9)	C24A—C23A—Fe1	71.2 (10)
C13—C14—H14A	109.5	C22A—C23A—H23B	125.6
C13—C14—H14B	109.5	C24A—C23A—H23B	125.6
H14A—C14—H14B	109.5	Fe1—C23A—H23B	126.1
C13—C14—H14C	109.5	C23A—C24A—C25A	107.5 (11)
H14A—C14—H14C	109.5	C23A—C24A—Fe1	68.9 (10)
H14B—C14—H14C	109.5	C25A—C24A—Fe1	70.0 (14)
C13—C15—H15A	109.5	C23A—C24A—H24B	126.3
C13—C15—H15B	109.5	C25A—C24A—H24B	126.3
H15A—C15—H15B	109.5	Fe1—C24A—H24B	126.4
C13—C15—H15C	109.5	C24A—C25A—C26A	107.9 (11)
H15A—C15—H15C	109.5	C24A—C25A—Fe1	70.2 (13)
H15B—C15—H15C	109.5	C26A—C25A—Fe1	68.6 (15)
C13—C16—H16A	109.5	C24A—C25A—H25B	126.0
C13—C16—H16B	109.5	C26A—C25A—H25B	126.0
H16A—C16—H16B	109.5	Fe1—C25A—H25B	126.7
C13—C16—H16C	109.5	C22A—C26A—C25A	108.1 (11)
H16A—C16—H16C	109.5	C22A—C26A—Fe1	68.9 (13)
H16B—C16—H16C	109.5	C25A—C26A—Fe1	71.2 (15)
C8A—C7A—C12A	118.2 (12)	C22A—C26A—H26B	125.9
C8A—C7A—N1	123 (2)	C25A—C26A—H26B	125.9
C12A—C7A—N1	119 (2)	Fe1—C26A—H26B	125.5

### Hydrogen-bond geometry (Å, º)

**Table:**

*D*—H···*A*	*D*—H	H···*A*	*D*···*A*	*D*—H···*A*
O2—H1*O*2···O1^i^	0.80 (4)	1.91 (5)	2.699 (4)	166 (5)

Symmetry code: (i) −*x*+1/2, *y*−1/2, −*z*+1/2.
